# Potent PPARα Activator Derived from Tomato Juice, 13-oxo-9,11-Octadecadienoic Acid, Decreases Plasma and Hepatic Triglyceride in Obese Diabetic Mice

**DOI:** 10.1371/journal.pone.0031317

**Published:** 2012-02-09

**Authors:** Young-il Kim, Shizuka Hirai, Tsuyoshi Goto, Chie Ohyane, Haruya Takahashi, Taneaki Tsugane, Chiaki Konishi, Takashi Fujii, Shuji Inai, Yoko Iijima, Koh Aoki, Daisuke Shibata, Nobuyuki Takahashi, Teruo Kawada

**Affiliations:** 1 Laboratory of Molecular Function of Food, Division of Food Science and Biotechnology, Graduate School of Agriculture, Kyoto University, Kyoto, Japan; 2 Chiba Prefectural Agriculture and Forestry Research Center, Chiba, Japan; 3 Nippon Del Monte Corporation, Gunma, Japan; 4 Kazusa DNA Research Institutes, Chiba, Japan; Governmental Technical Research Centre of Finland, Finland

## Abstract

Dyslipidemia is a major risk factor for development of several obesity-related diseases. The peroxisome proliferator-activated receptor α (PPARα) is a ligand-activated transcription factor that regulates energy metabolism. Previously, we reported that 9-oxo-10,12-octadecadienoic acid (9-oxo-ODA) is presented in fresh tomato fruits and acts as a PPARα agonist. In addition to 9-oxo-ODA, we developed that 13-oxo-9,11-octadecadienoic acid (13-oxo-ODA), which is an isomer of 9-oxo-ODA, is present only in tomato juice. In this study, we explored the possibility that 13-oxo-ODA acts as a PPARα agonist *in vitro* and whether its effect ameliorates dyslipidemia and hepatic steatosis *in vivo*. *In vitro* luciferase assay experiments revealed that 13-oxo-ODA significantly induced PPARα activation; moreover, the luciferase activity of 13-oxo-ODA was stronger than that of 9-oxo-ODA and conjugated linoleic acid (CLA), which is a precursor of 13-oxo-ODA and is well-known as a potent PPARα activator. In addition to *in vitro* experiment, treatment with 13-oxo-ODA decreased the levels of plasma and hepatic triglycerides in obese KK-Ay mice fed a high-fat diet. In conclusion, our findings indicate that 13-oxo-ODA act as a potent PPARα agonist, suggesting a possibility to improve obesity-induced dyslipidemia and hepatic steatosis.

## Introduction

Obesity is a major risk factor for chronic diseases including diabetes, cardiovascular diseases, and hypertension [1]–[4]. Dyslipidemia, in particular, is a direct risk factor for arteriosclerosis, and for liver cirrhosis, and may be partially due to the dysfunction of lipid metabolism in the liver. Therefore, to prevent or reduce arteriosclerosis and cirrhosis, it is important to ameliorate the dysfunction of hepatic lipid metabolism dysfunction.

Peroxisome proliferator-activated receptors (PPARs) are ligand-activated transcription factors and members of the nuclear hormone receptor superfamily, which regulate energy homeostasis (glucose and lipid metabolisms), inflammation, proliferation, and differentiation [5]–[9]. In particular, PPARα acts as a master regulator of fatty acid oxidation by controlling the transcription of its target genes [10], [11]. Consistent with this function, PPARα is mainly expressed in tissues with high lipid catabolic capacities, such as the liver, skeletal muscle, and brown adipose tissue [7], [12]. It has been reported that the activation of PPARα enhances fatty acid oxidation in the liver and decreases the levels of circulating and cellular lipids in obese diabetic patients [9], [13]. Therefore, the regulation of PPARα activity is one of the most important means of managing chronic disease related to dysfunction in lipid metabolism in the liver.

During the past decade, numerous studies have shown that endogenous and naturally occurring biological molecules, including fatty acids and fatty acid-derivatives, serve as PPARα agonists [14], [15]. In particular, conjugated linoleic acid (CLA) is well known as a potent PPARα agonist [16] and treatment with CLA actually increases the catabolism of lipids in the liver in rodents [17]. However, the effects of CLA derivatives on PPARα remain unclear.

Recently, we reported that a particular CLA derivative, 9-oxo-10,12-octadecadienoic acid (9-oxo-ODA), is present in fresh tomato fruit, and serves as a PPARα agonist [18]. In mouse primary hepatocytes, 9-oxo-ODA enhanced fatty acid oxidation via PPARα activation and consequently inhibited triglyceride accumulation [18]. Interestingly, we developed that processed products such as tomato juice contain 13-oxo-9,11-octadecadenoic acid (13-oxo-ODA), an isomer of 9-oxo-ODA, which was not present in fresh tomato fruit [19].

In this study, we explored whether 13-oxo-ODA acts as a PPARα agonist *in vitro* and ameliorates dyslipidemia and hepatic steatosis *in vivo*. Treatment with 13-oxo-ODA activated PPARα in mouse primary hepatocytes. Furthermore, treatment of obese diabetic KK-Ay mice with 13-oxo-ODA suppressed the increase in plasma and hepatic triglyceride (TG) levels resulting from a high-fat diet (HFD), through PPARα activation in peripheral tissues such as the liver and skeletal muscle. In addition, 13-oxo-ODA treatment decreased the plasma glucose level and increased glucose the tolerance ability in the 13-oxo-ODA-fed mice, as has been shown for other PPARα agonists. These findings indicate that 13-oxo-ODA has a possibility to improve the disorder of lipid and carbohydrate metabolism, via PPARα activation.

## Materials and Methods

### Plant materials and chemicals

The compound 13-oxo-9,11-ODA was synthesized by Shinsei Chemical Company, Ltd. (Osaka, Japan), and 9,11-octadecadienic acid (conjugated linoleic acid; CLA) was purchased from Cayman Chemical (MI, U.S.A). All other chemicals were purchased from Sigma (MO, U.S.A), Wako (Osaka, Japan), or Nacalai Tesque (Kyoto, Japan), and were guaranteed to be of reagent grade or tissue-culture grade.

### Luciferase assay

Luciferase assays were performed as previously described, using a GAL4/PPAR chimera system [20], [21]. We transfected p4xUASg-tk-luc (a reporter plasmid), pM-hPPARα (an expression plasmid for a chimera protein for the GAL4 DNA-binding domain and each human PPAR-ligand-binding domain), and pRL-CMV (an internal control for normalizing transfection efficiency) into monkey CV1 kidney cells by using Lipofectamine (Invitrogen Corp.), according to manufacturer's protocol. Luciferase activity was assayed using the dual luciferase system (Promega, MO, U.S.A) according to the manufacturer's protocol.

### Preparation of mouse primary hepatocytes

Mouse hepatocytes were prepared as previously described [18]. Briefly, C57/BL/6J male mice were anesthetized with intraperitoneal administration of Nembutal and the liver was perfused with liver perfusion medium (Invitrogen Corp.), followed by liver digestion medium (Invitrogen Corp.). Hepatocytes were dispersed in hepatocyte wash medium (Invitrogen Corp.) by dissection and gentle shaking. After filtration through a 100-µm nylon mesh filter, hepatocytes were isolated by repeated centrifugation at 50 *g* for 3 min (3 times). The isolated hepatocytes were cultured in type-1 collagen-coated 12-well plates (Iwaki, Chiba, Japan). After 5-h incubation at 37°C in 5% CO_2_ atmosphere, the hepatocytes were used for mRNA quantification assay.

### Animal experiments

Male KK-Ay mice, a useful model of obesity and diabetes [22], were purchased from CLEA Japan (Tokyo, Japan). The mice were kept in individual cages in a temperature-controlled room at 24±1°C and maintained under a constant 12-h light/dark cycle. All animal experiments were approved by Kyoto University Animal Care Committee (approval ID: No. 22–53).

To determine the effects of 13-oxo-ODA on the development of diabetic conditions, we used 4-week-old mice. The mice were maintained for 5 days on a standard diet and then divided into 3 groups of similar average body weight. Each group was maintained on 60% HFD (D12492; Research Diets, NJ, USA) or on HFD containing 0.02% (w/w) or 0.05% 13-oxo-ODA for 4 weeks. The energy intake of all the mice was adjusted by pair feeding. The energy intake of all the mice was adjusted by pair feeding. Thus, the levels of food intake of each group was similar (average food intakes were 3.42±0.05, 3.30±0.09, and 3.53±0.02 g/day in the groups fed control HFD, 0.02% 13-oxo-ODA, and 0.05% 13-oxo-ODA, respectively).

An oral glucose tolerance test (OGTT) was performed on the KK-Ay mice fed the experimental diet for 3 weeks [23]. For OGTT, glucose (1.5 g/kg body weight) was administered orally after overnight fasting, and blood samples collected from the tail vein before and 15, 30, 60, 90, and 120 min after the administration.

During the 4 weeks of the treatment period, the rectal temperature of all the mice was also measured using a thermometer probe (T&D Corp., Nagano, Japan).

At the end of the treatment period, anesthetized mice were killed by cervical dislocation after overnight fasting, and blood samples were collected. Plasma TG and glucose levels were determined by the TG E-test and glucose CII-test (Wako), respectively. Plasma insulin and adiponectin concentrations were measured using an ultrasensitive mouse insulin kit (Morinaga Institute of Biological Science, Yokohama, Japan) and ELISA kits (BD Bioscience, CA, USA), respectively. All kits were used in accordance with the manufacturer's protocols.

### Histological analysis of liver

Liver samples were removed from each animal and fixed in 10% formaldehyde/PBS. The fixed samples were embedded in a tissue-freezing medium (Tissue-Tek OCT compound; IN, USA) and frozen in acetone cooled with liquid nitrogen. Cryostat sections of 10-µm thickness were prepared at −20°C, fixed in 50% ethanol/water for 5 min, and then stained with Oil Red O. The sections were counterstained with Mayer's hematoxylin.

### Measurements of TG contents in tissues

To determine liver and skeletal muscle TG contents, TGs were extracted from those tissues with chloroform/methanol by the Bligh Dyer method [24]. TG concentration was measured using the TG E-test (Wako).

### Quantification of mRNA expression levels

Total RNA was prepared from primary hepatocytes, as well as liver and skeletal muscle, using Sepasol (Nacalai Tesque), according to the manufacturer's protocols. Using M-MLV reverse transcriptase (Invitrogen, Corp.), total RNA was reverse-transcribed using a thermal cycler (Takara PCR Thermal Cycler SP: Takara Bio Inc., Shiga, Japan). To determine mRNA expression levels, real-time quantitative RT-PCR analysis was performed with a Light Cycler System (Roche Diagnostics) using SYBR green fluorescence signals, as described previously [20], [25].

The oligonucleotide primer sets of mouse 36B4, PPARα target genes, and TG synthesis related genes were designed using a PCR primer selection program at the website of the Virtual Genomic Center from the GenBank database as follows: mouse *CPT1a* (Fwd: 5′-ctcagtgggagcgactcttca-3′; Rev: 5′-ggcctctgtggtacacgacaa-3′), mouse *CPT1b* (Fwd: 5′-ctgttaggcctcaacaccgaac-3′; Rev: 5′-ctgtcatggctaggcggtacat-3′), mouse *AOX* (Fwd: 5′-gcaccattgccattcgataca-3′; Rev: 5′-acggctattctcacagcagtgg-3′), mouse *FAT/CD36* (Fwd: 5′-gatgtggaacccataactggattcac-3′; Rev: 5′-ggtcccagtctcatttagccacagt-3′), mouse *ACS* (Fwd: 5′- acatccacgtgtatgagttctacgc-3′; Rev: 5′-agtagacgaagttctcacggtcgat-3′), mouse *UCP2* (Fwd: 5′-cactttccctctggataccgc-3′; Rev: 5′-gatcccttcctctcgtgcaat-3′). mouse *SREBP1c* (Fwd: 5′-ggagccatggattgcacatt-3′; Rev: 5′-gccagagaagcagaagagaag-3′), mouse *ABCA1* (Fwd: 5′-ggacttggtaggacggaacct-3′; Rev: 5′-tcctcatcctcgtcattcaaa-3′), mouse *ABCG1* (Fwd: 5′-aagacctgcactgcgacatc-3′; Rev: 5′-tggtcacaatctctgctttg-3′), mouse *FAS* (Fwd: 5′-tgggttctagccagcagagt-3′; Rev: 5′-accaccagagaccgttatgc-3′), and mouse *36B4* as an internal control (Fwd: 5′-tgtgtgtctgcagatcgggtac-3′; Rev: 5′-ctttggcgggatttagtcgaag-3′). All data indicating mRNA expression levels are presented as a ratio relative to a control in each experiment.

### Immunoblotting assay

Immunoblotting was carried out as previously described [18]. The anti-mouse AOX antibody was obtained from Abcam Corp. (MA, U.S.A). Anti-mouse β-actin and horseradish peroxidase (HRP)-conjugated anti-rabbit IgG was purchased from Santa Cruz Biotechnology Inc. (CA, U.S.A). Protein bands were detected by chemiluminescence using an enhanced chemiluminescence system (NEN Lifescience Products) in accordance with the manufacturer's instructions.

### Statistical analyses

Data are presented as mean ± SEM. The data were assessed for statistical significance by 1-way ANOVA and Dunnet's multiple comparison tests. Differences were considered significant when *p* was <0.05.

## Results

### 13-oxo-ODA serves as PPARα ligand

**Figure 1:**
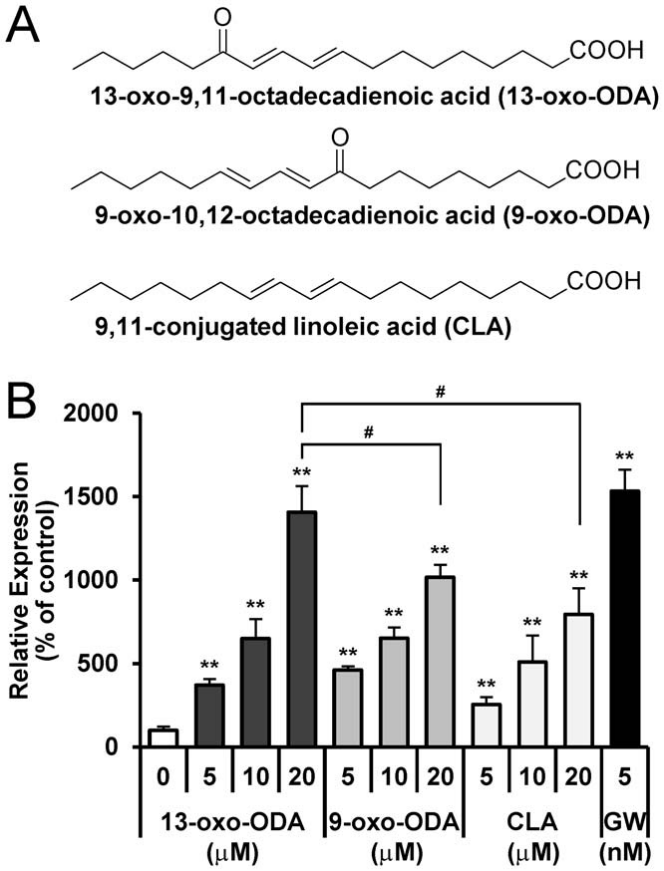
Effects of 13-oxo-ODA on PPARα activation determined by luciferase assay and using mouse primary hepatocytes. (A) Chemical structures of 13-oxo-ODA, 9-oxo-ODA, and CLA. (B) A reporter plasmid (p4xUASg-tk-luc) and an expression vector for a GAL4 PPARα chimeric protein (pM-hPPARα) were transfected into CV1 cells together with an internal control reporter plasmid (pRL-CMV). Twenty-four hours after the transfection, the cells were treated with synthesized CLA or 13-oxo-ODA for 24 h. GW7647 (5 nM), which is a PPARα-specific agonist, was used as a positive control. Luciferase activity was measured using a dual luciferase system. The activity of a vehicle control was set at 100%, and the relative luciferase activities are presented as fold induction with respect to that of the vehicle control. Data are presented as mean ± SEM (n = 4−5). **; *p*<0.01 *versus* control. #; *p*<0.05 compared between indicated groups.

First, we investigated whether 13-oxo-ODA (Fig. 1A) activated PPARα in a luciferase ligand assay by using the GAL4/PPAR chimera system and CV1 cells, and found that treatment with 13-oxo-ODA increased luciferase activity in a dose-dependent manner as well as that of GW7647, a synthetic PPARα agonist (Fig. 1B). Furthermore, we compared the PPARα reporter activities in response to 13-oxo-ODA vs. 9-oxo-ODA (Fig. 1A), which is an isomer of 13-oxo-ODA [19] and acts as a PPARα activator [18]. Interestingly, the luciferase activity generated by 13-oxo-ODA was stronger than that of 9-oxo-ODA (Fig. 1B). Moreover, the luciferase activity of 13-oxo-ODA also showed higher than that of CLA, which is a precursor of 13-oxo-ODA [26] and is a well-known PPARα agonist (Fig. 1).

To elucidate whether 13-oxo-ODA induces PPARα activation in intact cells, we examined the effects of 13-oxo-ODA on mRNA expression levels of PPARα target genes in mouse primary hepatocytes. As expected, treatment with 13-oxo-ODA for 24 h significantly increased the mRNA expression levels of carnitine-palmitoyl transferase 1a (*CPT1a*; subtype of liver), acyl-CoA oxidase (*AOX*), fatty acid translocase (*FAT*), acyl-CoA synthase (*ACS*), and uncoupling protein 2 (*UCP2*) in the primary hepatocytes similar to the treatment with CLA (Fig. S1). Furthermore, to investigate whether these effects of 13-oxo-ODA depend on PPARα, we coadministered 13-oxo-ODA and GW6471, a PPARα-specific antagonist [27]. The increase in those mRNA expression levels by 13-oxo-ODA was abolished by GW6471, although the basal levels of these mRNA expressions remained unchanged. These findings indicate that 13-oxo-ODA, a CLA-derivative, activates PPARα in hepatocytes.

### 13-oxo-ODAtreatment improved dyslipidemia and lowering TG content in the liver and skeletal muscle of HFD-fed KK-Ay mice

**Figure 2:**
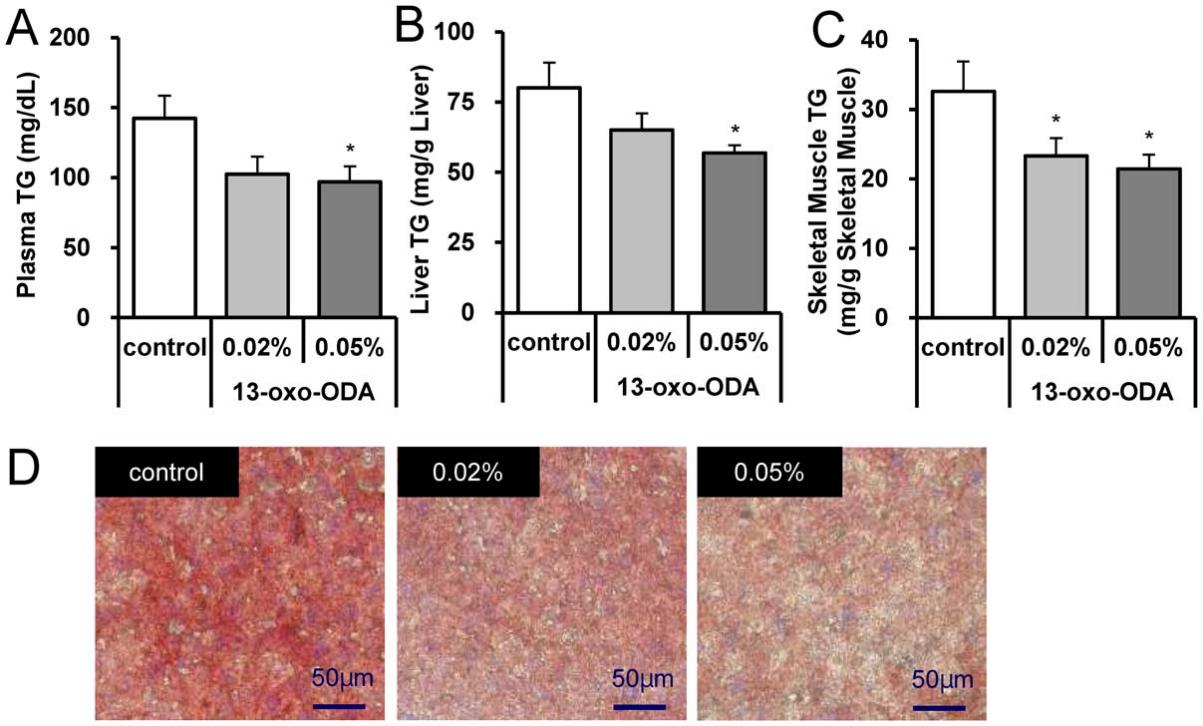
Effects of 13-oxo-ODA on plasma and hepatic TG contents in HFD-fed KK-Ay mice. Plasma TG concentration (A) and hepatic TG content (B) at the end of treatment period. Data are presented as mean ± SEM (n = 4−5). *; *p*<0.05 *versus* control. (C) Isolated livers were fixed in 10% formalin/PBS for more than 24 h and then embedded. Liver sections were cut into 10 µm sections. The liver sections were stained with Oil Red O and Mayer's hematoxylin. A bar shown in each photograph indicates 50 µm.

To determine *in vivo* effects of 13-oxo-ODA on the development of obesity-related metabolic dysfunction, KK-Ay mice were fed a HFD containing 0.02% or 0.05% synthesized 13-oxo-ODA for 4 weeks. The body weights showed no significant differences between the control HFD-fed and 13-oxo-ODA-fed groups (The average body weights were 36.2±0.51, 36.3±0.75, and 37.1±0.58, in the groups fed control HFD, 0.02% 13-oxo-ODA, and 0.05% 13-oxo-ODA, respectively). In addition of body weights, the weights of white adipose tissues, liver, skeletal muscle, and brown adipose tissue showed no significant differences between the control HFD-fed and 13-oxo-ODA-fed groups (data not shown). However, plasma TG concentration significantly decreased in the 0.05% 13-oxo-ODA-fed mice (Fig. 2A), as did TG content in the liver and skeletal muscle (Fig. 2B and C). Furthermore, Oil red O staining of the liver sections supported this finding, by showing that TG accumulation was suppressed in the liver of the 13-oxo-ODA-treated mice (Fig. 2D) but was present at high levels in the liver of the control mice. The suppression was more apparent in the 0.05% 13-oxo-ODA-containing HFD-fed mice than in the 0.02% 13-oxo-ODA-containing HFD-fed mice, suggesting a dose effect. Thus these findings indicate that treatment with 13-oxo-ODA suppresses the obesity-induced TG accumulation in the liver and skeletal muscle.

### 13-oxo-ODA treatment induced expression of PPARα target genes in the liver and skeletal muscle

**Figure 3:**
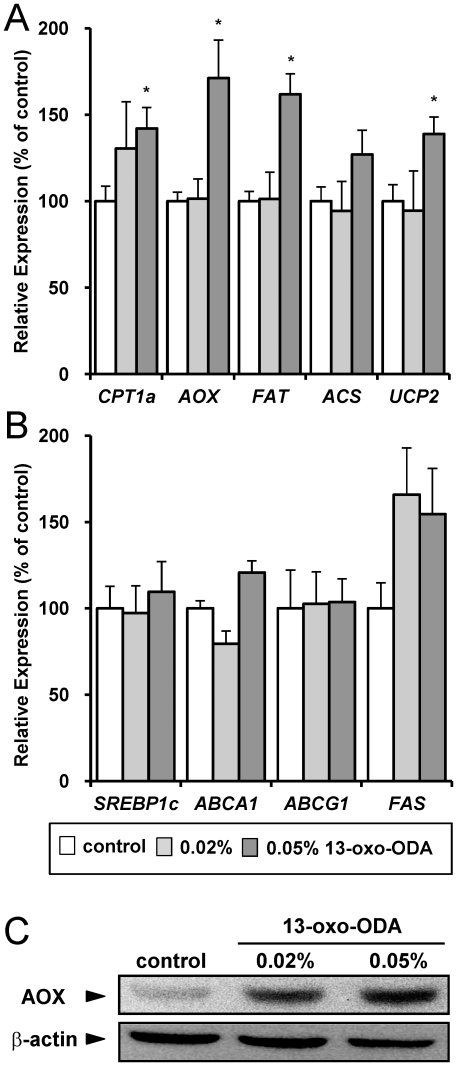
Effects of 13-oxo-ODA on mRNA expression levels of genes involved in lipid metabolism in the liver of HFD-fed KK-Ay mice. The mRNA expression levels of genes involved in fatty acid metabolism (*CPT1a*, *AOX*, *FAT*, *ACS*, and *UCP2*; Fig. 3A) and in TG synthesis (*SREBP1c*, *ABCA1*, *ABCG1*, and *FAS*; Fig. 3B) in the liver of control and 13-oxo-ODA-fed mice were quantified by real-time PCR. The relative amount of each transcript was normalized to the amount of the *36B4* transcript. The expression levels in the vehicle control are set at 100% and the relative expression levels are presented as fold induction with respect to that in the vehicle control. (C) Protein expression of AOX and β-actin in the liver of control and 13-oxo-ODA-fed mice were compared by immunoblotting assay. The same amounts of protein (25 µg/lane) were loaded and blotted onto PVDF membranes. The membranes were sequentially treated with primary antibodies as indicated and secondary antibodies conjugated with HRP. The enhanced chemiluminescence system was used for visualization of membranes. Data are presented as mean ± SEM (n = 6−8). *; *p*<0.05 *versus* control.

**Figure 4:**
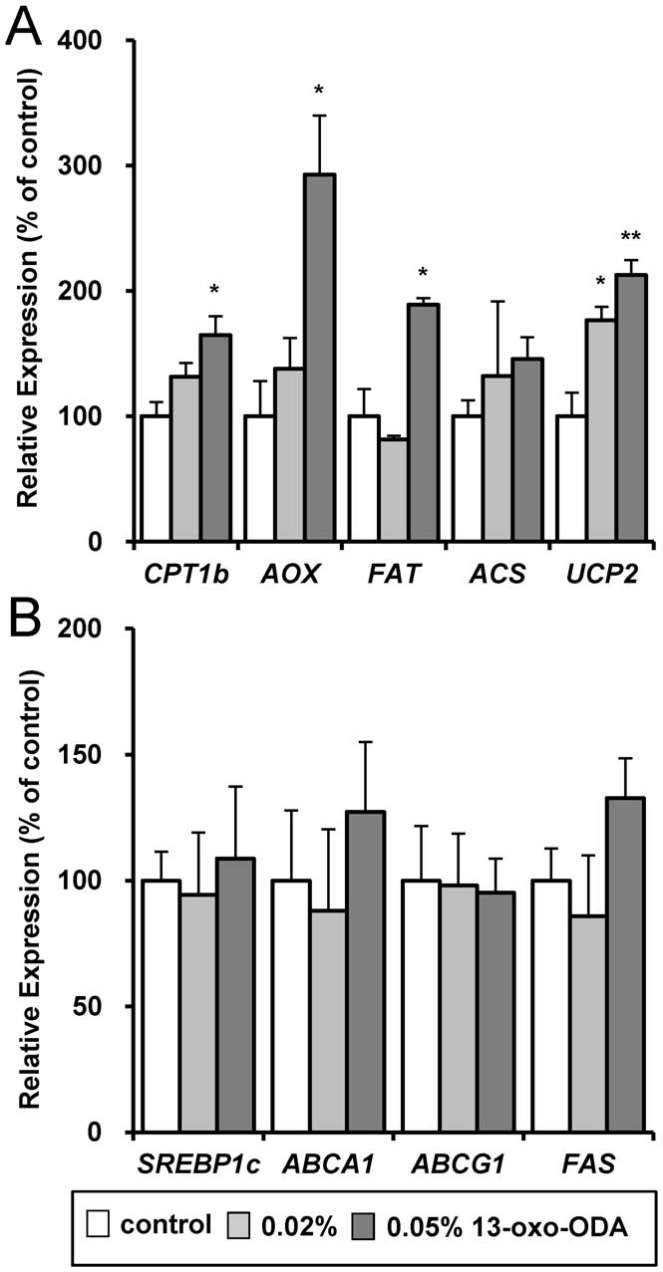
Effects of 13-oxo-ODA on mRNA expression levels of genes involved in lipid metabolism in the skeletal muscle of HFD-fed KK-Ay mice. The mRNA expression levels of genes involved in fatty acid metabolism (*CPT1b*, *AOX*, *FAT*, *ACS*, and *UCP2*; Fig. 4A) and in TG synthesis (*SREBP1c*, *ABCA1*, *ABCG1*, and *FAS*; Fig. 4B) in the skeletal muscle of control and 13-oxo-ODA-fed mice were quantified as described before. Data are presented as mean ± SEM (n = 6−8). *; *p*<0.05, **; *p*<0.01 *versus* control.

To determine the mechanism by which 13-oxo-ODA decreased plasma and hepatic TG concentrations, we measured mRNA expression levels of genes involved in fatty acid metabolism in the liver. The mRNA expression levels of *CPT1a*, *AOX*, *FAT*, *ACS*, and *UCP2* were increased in the liver by the 0.05% 13-oxo-ODA treatment (Fig. 3A). In contrast, treatment with 13-oxo-ODA didn't affect mRNA expression levels of genes involved in TG synthesis such as sterol regulatory element-binding protein 1c (*SREBP1c*), ATP-binding cassette sub-family A member 1 (*ABCA1*), *ABCG1*, and fatty acid synthase (*FAS*) (Fig. 3B). Additionally, the expression level of the AOX protein was increased by treatment with 13-oxo-ODA (Fig. 3C). Furthermore, we measured the effect of 13-oxo-ODA on these mRNA expression levels in the skeletal muscle, in which PPARα is highly expressed same as the liver. 13-oxo-ODA treatment induced mRNA expressions of *CPT1b* (subtype of skeletal muscle), *AOX*, *FAT*, *ACS*, and *UCP2* in the skeletal muscle (Fig. 4A), whereas mRNA expressions of *SREBP1c*, *ABCA1*, *ABCG1*, and *FAS* didn't change by 13-oxo-ODA treatment (Fig. 4B). Thus these findings indicate that treatment with 13-oxo-ODA induced the mRNA expression levels of genes involved in fatty acid metabolism, but did not affect TG synthesis-related genes expression in the liver and skeletal muscle.

### 13-oxo-ODA treatment increased the rectal temperature

**Figure 5:**
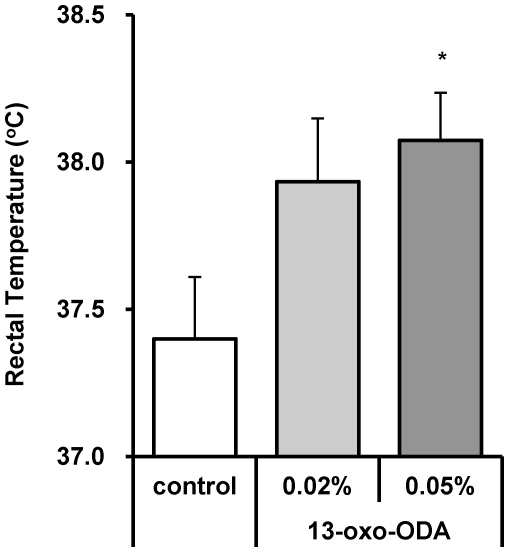
Effects of 13-oxo-ODA on rectal temperature in HFD-fed KK-Ay mice. At 4 weeks of the treatment period, the rectal temperature of all mice was measured using a thermometer probe. Data are presented as mean ± SEM (n = 6−8). *; *p*<0.05 *versus* control.

A previous study showed that rectal temperature increases concomitantly with energy expenditure, and that PPARα activation increases rectal temperature [28]; therefore we measured rectal temperature of the mice. In this study, the rectal temperature was 0.67°C higher in the 0.05% 13-oxo-ODA-treated mice than in the control mice (Fig. 5). Together, these findings indicate that 13-oxo-ODA increases the mRNA expression levels of PPARα target genes in the liver and skeletal muscle, thereby increasing fatty acid oxidation in these tissues.

### 13-oxo-ODA improved carbohydrate metabolism in HFD-fed KK-Ay mice

**Figure 6:**
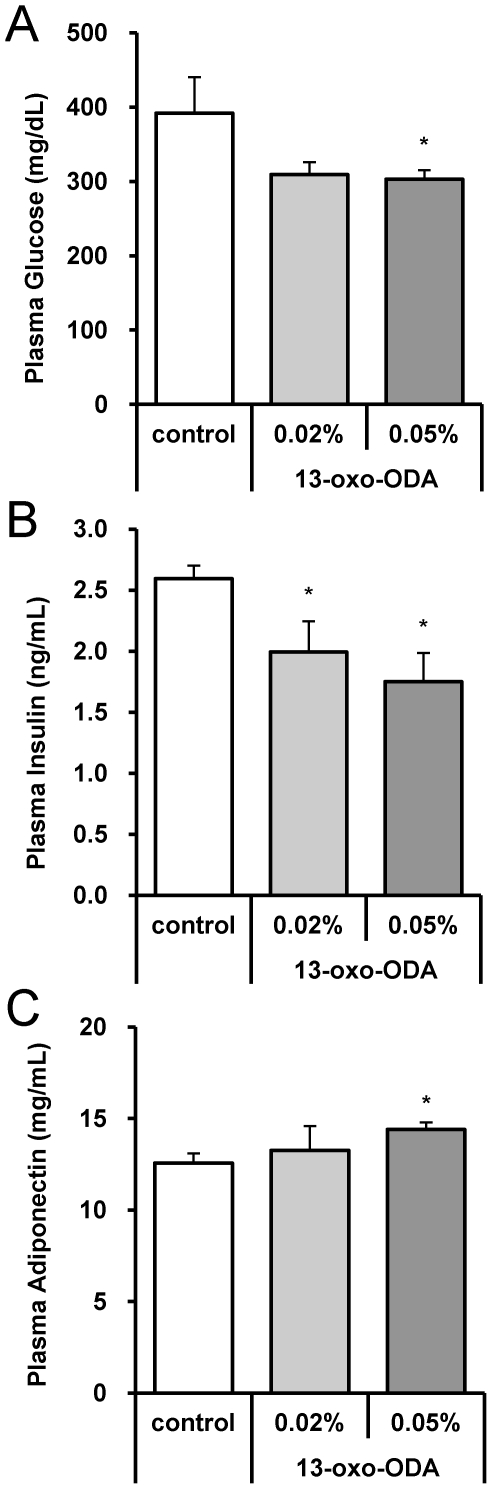
Effects of 13-oxo-ODA on carbohydrate metabolism in HFD-fed KK-Ay mice. Fasting plasma glucose (A), insulin (B), and adiponectin (C) in the KK-Ay mice fed HFD with or without 13-oxo-ODA for 4 weeks. Each bar represents the mean ± SEM (n = 6−8). *; *p*<0.05 *versus* control.

**Figure 7:**
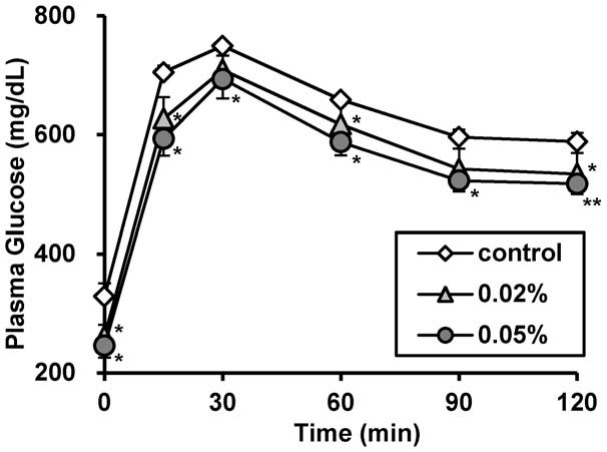
Effects of 13-oxo-ODA on glucose tolerance in HFD-fed KK-Ay mice. OGTT was performed at 3 weeks period. For OGTT, glucose (1.5 g/kg body weight) was orally administered after an overnight fasting. Each bar represents the mean ± SEM (n = 6−8). ◊, vehicle; ▴,0.02% 13-oxo-ODA; •, 0.05% 13-oxo-ODA. *; *p*<0.05, **; *p*<0.01 *versus* control.

Finally, to investigate the effects of 13-oxo-ODA on carbohydrate metabolism *in vivo*, we examined plasma levels of glucose in the 13-oxo-ODA-treated mice. As shown in Figs. 6A and B, 4-week HFD feeding resulted in high plasma levels of glucose and insulin, suggesting that the mice showed indications of diabetes. However, 13-oxo-ODA treatment significantly decreased both plasma glucose and insulin concentrations (22% and 32% decreases in the 0.02% and 0.05% 13-oxo-ODA-fed mice, respectively). In addition, the concentration of plasma adiponectin, an adipocytokine that improves insulin resistance [29], increased in the 13-oxo-ODA-fed mice (Fig. 6C). Furthermore OGTT showed that the plasma glucose concentration in the 0.05% 13-oxo-ODA-fed mice decreased more rapidly than that in the control mice (Fig. 7).

## Discussion

PPARα is activated by endogenous agonists, which include fatty acids and their derivatives such as eicosanoids and oxidized fatty acids, as well as by synthetic compounds [6], [30]–[32]. In this study, we explored whether the oxidized fatty acid, 13-oxo-ODA, which is an isomer of 9-oxo-ODA that is present in tomato, acts as a PPARα agonist [18]. Luciferase reporter assays revealed that 13-oxo-ODA induced PPARα activation in a dose-dependent manner. Furthermore, 13-oxo-ODA increased mRNA expression levels of PPARα target genes in mouse primary hepatocytes. These activities of 13-oxo-ODA were similar to those of 9-oxo-ODA (data not shown). In addition, the activities of 13-oxo-ODA were similar to those of CLA, which is considered a functional nutrient that improves abnormalities of lipid metabolism by activating PPARα [16] and increasing fatty acid oxidation [33]. Thus, our findings suggest that 13-oxo-ODA, as a PPARα agonist, is also valuable for control of lipid metabolism, similar to 9-oxo-ODA and CLA.

It has previously been established that activation of PPARα enhances fatty acid oxidation in the liver and decreases the levels of circulating and hepatic lipids in obese diabetic mice [9], [13]. When we examined the *in vivo* effects of 13-oxo-ODA in obese KK-Ay mice fed a HFD for 4 weeks, we found that treatment with 13-oxo-ODA increased the mRNA expression levels of PPARα target genes such as *CPT1*, *AOX*, *CD36*, *ACS*, and *UCP2* in both the liver and skeletal muscle. Furthermore, the rectal temperature, which indicates energy metabolism [28], was significantly higher in the 13-oxo-ODA-treated mice than in the control mice. Moreover, 13-oxo-ODA treatment suppressed increases plasma and hepatic TG in the obese KK-Ay mice fed HFD. However, 13-oxo-ODA didn't affect to the weights of body and WAT in this experiment. We think this period of treatment with 13-oxo-ODA (4weeks) may be too short to decrease the weights of body and WAT. Therefore, we need a longer treatment with 13-oxo-ODA for examining effects on weights of body and WAT, in addition to the decreases in hepatic and plasma levels of TG. These findings indicate that 13-oxo-ODA serves as a PPARα agonist both *in vivo* and *in vitro*, and suggested a possibility that 13-oxo-ODA is a beneficial food-derived compound for controlling plasma and hepatic levels of TG under obese and diabetic conditions.

It has also previously been shown that PPARα plays an important role in carbohydrate metabolism in addition to lipid metabolism [34], by enhancing fatty acid clearance from insulin-sensitive tissues, such as the liver and skeletal muscle [35]. Indeed, in this study, the treatment with 13-oxo-ODA also decreased the levels of plasma glucose and insulin. Furthermore, it has recently been revealed that PPARα agonists directly and transcriptionally increase adiponectin production via adipose PPARα activation [36], [37]. It has been well-known that adiponectin improves insulin resistance [38]. In fact, the plasma adiponectin level in 13-oxo-ODA-treated mice significantly increased. Thus the increase in plasma adiponectin level induced by the 13-oxo-ODA treatment may contribute to the improvement of insulin resistance.

A number of previous studies have shown that tomato products, especially tomato juices, contain various beneficial natural compounds that change hepatic lipid metabolism [39]–[41]. These beneficial effects of tomato are generally attributed to different compounds such as carotenoids, vitamins, and flavonoids, but the actual bioactive compound and molecular mechanisms involved in the beneficial effects remain unclear. In this study, we revealed 13-oxo-ODA as a PPARα activator decreasing plasma and hepatic levels of TG. Given that tomato is the most widely produced crop worldwide (136 million tons or more a year; FAO statics, 2008) and that large amounts of tomatoes are preprocessed and consumed as industrial products worldwide, it is serendipitous that this bioactive compound is present in processed tomato products, in particular.

In conclusion, this study indicated that 13-oxo-ODA acts as an agonist for PPARα not only *in vitro* but also *in vivo* where it activated hepatic PPARα, resulting in suppresses of obesity-induced plasma and hepatic levels of TG. These findings suggest a possibility that 13-oxo-ODA is a food-derived functional compound that can regulate hepatic lipid metabolism.

## Supporting Information

Figure S1 **Effects of 13-oxo-ODA on PPARα target gene expressions in mouse primary hepatocytes.** mRNA expression levels of *CPT1a*, *AOX*, *FAT*, *ACS*, and *UCP2* in mouse primary hepatocytes treated with 20 µM CLA or 13-oxo-ODA and/or 5 µM GW6471 for 24 h. GW6471 is a PPARá-specific antagonist. The amounts of mRNAs were quantified by real-time PCR. The relative amount of each transcript was normalized to the amount of the *36B4* transcript. The activity of a vehicle control was set at 100% and the relative expression levels are presented as fold induction with respect to that in the vehicle control. Data are presented as mean ± SEM (n = 4). *; *p*<0.05, **; *p*<0.01 *versus* control. #; *p*<0.05 compared between indicated groups.
(TIF)
