# Supplementary figures and images for: Potent PPARα Activator Derived from Tomato Juice, 13-oxo-9,11-Octadecadienoic Acid, Decreases Plasma and Hepatic Triglyceride in Obese Diabetic Mice
Source: PLoS One. 2012 Feb 9;7(2):e31317. doi: 10.1371/journal.pone.0031317 (PMC3276502; doi:10.1371/journal.pone.0031317)

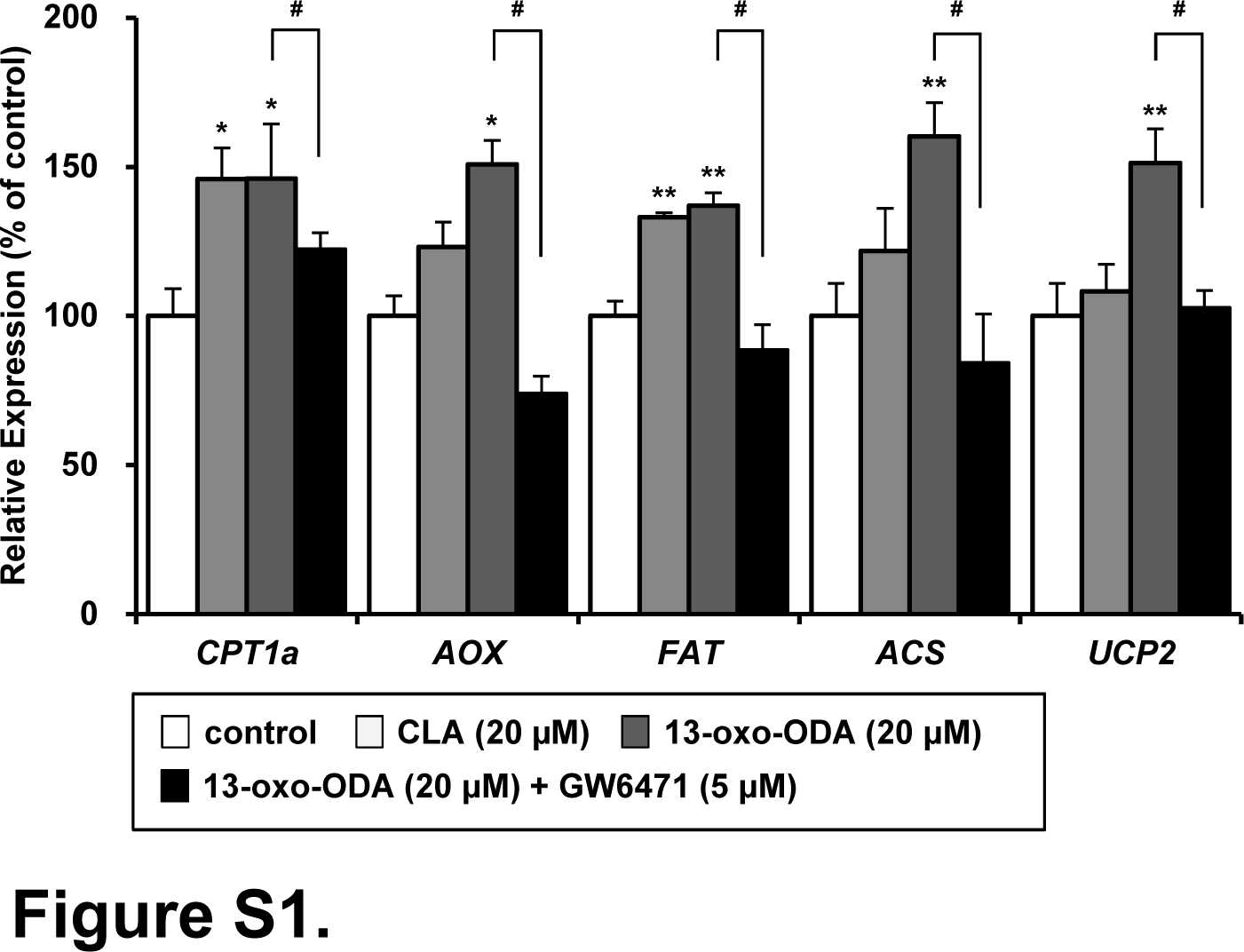

Supplement: Figure S1 — Effects of 13-oxo-ODA on PPARα target gene expressions in mouse primary hepatocytes. mRNA expression levels of CPT1a, AOX, FAT, ACS, and UCP2 in mouse primary hepatocytes treated with 20 µM CLA or 13-oxo-ODA and/or 5 µM GW6471 for 24 h. GW6471 is a PPARá-specific antagonist. The amounts of mRNAs were quantified by real-time PCR. The relative amount of each transcript was normalized to the amount of the 36B4 transcript. The activity of a vehicle control was set at 100% and the relative expression levels are presented as fold induction with respect to that in the vehicle control. Data are presented as mean ± SEM (n = 4). *; p<0.05, **; p<0.01 versus control. #; p<0.05 compared between indicated groups. (TIF) [file pone.0031317.s001.tif]
